# Comparison of Ten Metal-Doped LaFeO_3_ Samples on Photocatalytic Degradation of Antibiotics in Water under Visible Light: Role of Surface Area and Aqueous Phosphate Ions

**DOI:** 10.3390/molecules28093807

**Published:** 2023-04-29

**Authors:** Isabella Bolognino, Renato Pelosato, Giuseppe Marcì, Isabella Natali Sora

**Affiliations:** 1 Department of Engineering and Applied Sciences and INSTM, University of Bergamo, Viale Marconi 5, 24044 Dalmine, Italy; 2 “Schiavello Grillone” Photocatalysis Group, Department of Engineering, University of Palermo, Viale delle Scienze, 90128 Palermo, Italy

**Keywords:** heterogeneous photocatalysis, lanthanum ferrite, ciprofloxacin, oxytetracycline, perovskite

## Abstract

Doping semiconducting oxides, such as LaFeO_3_ (LF), with metallic elements is a good strategy to improve the performance of photocatalysts. In this study, LF and ten different nanopowders metal-doped at the La or Fe site of LaFeO_3_ were evaluated in the photocatalytic degradation of ciprofloxacin (CP) and oxytetracycline (OTC). The following metals were used in the doping (mol%) process of LF: Pd 3% and 5%; Cu 10%; Mg 5%, 10%, and 20%; Ga 10%; Y 10% and 20%; and Sr 20%. The doped samples were synthetized using a citrate auto-combustion technique. From the X-ray diffraction (XRD) data, only a single crystalline phase, namely an orthorhombic perovskite structure, was observed except for trace amounts of PdO in the sample with Pd 5%. The specific surface area (SSA) ranged from 9 m^2^ g^−1^ (Ga 10%) to 20 m^2^ g^−1^ (Mg 20%). SEM images show that all samples were constituted from agglomerates of particles whose sizes ranged from ca. 20 nm (Mg 20%) to ca. 100 nm (Pd 5%). Dilute aqueous solutions (5 × 10^−6^ M) prepared for both CP and OTC were irradiated for 240 min under visible-light and in the presence of H_2_O_2_ (10^−2^ M). The results indicate a 78% removal of OTC with Cu 10% doped LF in a phosphate buffer (pH = 5.0). The degradation of CP is affected by pH and phosphate ions, with 78% (in unbuffered solution) and 54% (in phosphate buffer, pH = 5.0) removal achieved with Mg 10% doped LF. The reactions follow a pseudo-first order kinetic. Overall, this study is expected to deepen the assessment of photocatalytic activity by using substrates with different absorption capacities on photocatalysts.

## 1. Introduction

Besides conventional wastewater treatment methods, the application of advanced oxidation processes (AOPs) has received widespread attention for decades. AOPs are based on the formation and reaction of reactive oxidation species (ROS), mostly generated by light-initiated reactions. Among AOPs, photocatalysis has attracted the interest of researchers because it represents an effective and sustainable technology that can help solve environmental management problems, particularly pollutants in wastewater [1,2]. In the photocatalytic process, a chemical reaction is activated or its rate is changed when a semiconductor photocatalyst is irradiated by light with an energy that matches or exceeds the band gap energy of the semiconductor, resulting in excited electron-hole (e^−^-h^+^) pairs [3]. The electrons are then promoted from the valence band (VB) to the conduction band (CB), while the holes remain in the VB. The electrons or holes interact with pollutants in the water, forming reaction intermediates. If the intermediate compounds are harmful, photocatalysis can be used to degrade them in turn. The presence of radiation in the visible light region of the electromagnetic spectrum is very attractive because it makes the operation of wastewater treatment plants less complicated and significantly reduces costs. In particular, visible light photocatalysis can be used as a complementary technique to conventional wastewater treatments (e.g., biological, chlorination, etc.) for “purified” effluent from the wastewater plant, which still contains recalcitrant pollutant molecules in very small concentrations (<1 μg/L).

Several studies have shown that LaFeO_3_-based semiconductors are promising visible-light and UV photocatalysts for aqueous reactions [4,5], often combined with a hydrogen peroxide reaction [6,7]. Recently, the effects of peroxydisulfate (PS) on LF/UV-A treatment was explored for the first time [8,9]. Furthermore, LaFeO_3_ is an effective photocatalyst for As(III) oxidation to As(V) in aqueous environments under UV-C [10]. LaFeO_3_-based oxides offer the advantages of LaFeO_3_ being a stable oxide p-type semiconductor with a band medium gap of 2.48 eV [10] and both low cost and non-toxic. These perovskite oxides have the general formula of ABO_3_, with A cation being a larger size than B. The ideal (cubic) perovskite structure consists of a three-dimensional system of vertex-sharing [BO_6_] octahedral, with A-site cations coordinated 12-fold in the cubo-octahedral cages. Frequently, perovskite oxides adopt a variant with lower symmetry than cubic. Different dopants may control phase transformation, regulate electrical conductivity, alter catalytic properties, or perform various other functions. The partial substitution of A or B cations gives rise to substituted compounds with the formula La_1−x_M_x_FeO_3_ or LaFe_1−x_M_x_O_3_. In cases where the charge of M is less than that of Fe, the oxygen atoms slightly shift toward the more charged cation, although the octahedral symmetry of Fe and M is preserved. Consequently, their structural stability and physical properties can remarkably be modified.

LaFeO_3_, like many ABO_3_ perovskite-type oxides, presents an extensive recombination rate for photogenerated electron-hole pairs [11]. Perovskite doping with metal elements can be a suitable strategy to increase its charge transport properties and decrease its rate of electron-hole recombination. However, in the literature, the effects of metal-doping several times on the La and Fe sites using equal experimental conditions have been seldom reported [12]. Phan et al. [13] reported that the crystallite sizes of Cu-doped LaFeO_3_ samples are smaller than those of LaFeO_3_ because Cu doping causes lattice distortion and suppresses the growth of large crystallites in the samples. The subsequent high degree of crystallinity with few defects helps to minimize the recombination of electron-hole pairs, leading to an enhanced efficiency of the photodegradation of organic dyes [14].

The literature has reported a strong affinity between La and phosphate and the consequent possibility of using LaFeO_3_ to effectively remove phosphate pollution. The absorption of phosphate consists in replacing its surface hydroxyl groups and forming mononuclear and binuclear complexes. Therefore, the absorption mechanism of surface-ligand exchange reactions occurs between phosphate and hydroxyl groups, and phosphate absorption can significantly increase the number of negative charges on the LaFeO_3_ surface [15].

The presence of pharmaceuticals in surface water bodies has generated great concern. Their risks to human health, such as water-related illnesses, are well-documented, particularly in relation to antimicrobial resistance [16]. Among antibiotics, oxytetracycline (OTC) and ciprofloxacin (CP) are most frequently detected in aquatic environments. OTC is widely used for the treatment of infections in both humans and animals and as a feed additive for promoting animal growth in the livestock and fish-farming industries [17]. CP is commonly used to treat bacterial infections, such as urinary tract infections and pneumonia [18]. Their properties (hydrophilicity and a stable ring structure) make them scarcely removable from the aqueous substrate using conventional wastewater treatment methods [19].

In this study, the effects of the partial substitution of La or Fe ions in three different La_1-x_A_x_FeO_3_ compounds and seven different LaFe_1-x_B_x_O_3_ compounds on the photocatalytic degradation of OTC and CP were investigated under visible-light irradiation and in the presence of H_2_O_2_. The addition of H_2_O_2_ increases the photodegradation rate of organic pollutants by removing surface-trapped electrons, thereby lowering the electron–hole recombination rate and increasing the efficiency of hole utilization for reactions such as OH^−^ + h^+^ → •OH [20].

## 2. Results and Discussion

### 2.1. Microstructural Characterization

**Figure 1:**
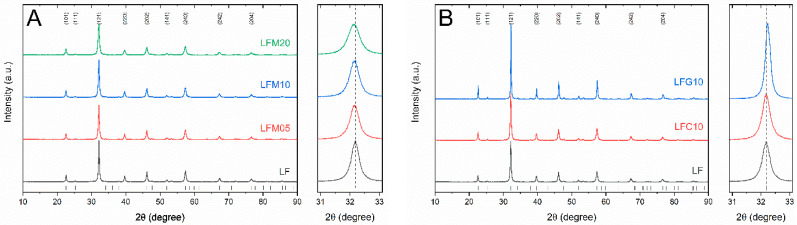
(**A**) X-ray diffraction patterns of LF, LFM05, LFM10, and LFM20; (**B**) diffraction patterns of LF, LFC10, and LFG10. On the right are enlargements of the (121) peak.

**Figure 2:**
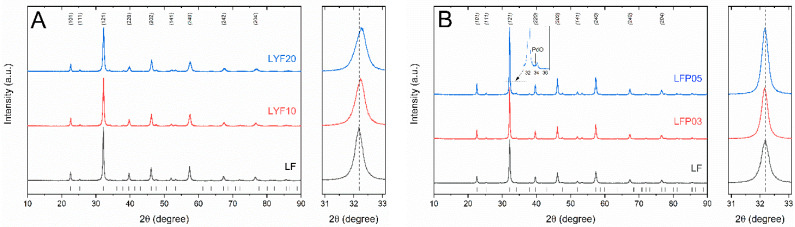
(**A**) X-ray diffraction patterns of LF, LFY10, and LFY20 are shown: the peaks are slightly shifted towards high angles, corresponding to cell size shrinking and increasing the amount of Y^3+^; (**B**) X-ray diffraction patterns of LF, LFP03, and LFP05. On the right are enlargements of the (121) peak.

The structural characterization of the as-prepared photocatalysts confirms that all the powders used in this study are single phases except for trace amounts of PdO in the LFP05 sample. All the compounds crystallized in a perovskite structure with an orthorhombic cell (space group *Pnma*) with lattice parameters close to those of LF (a = 5.5680(2) Å, b = 7.8561(3) Å, c = 5.5537(2) Å). X-ray diffraction patterns of the photocatalyst powders are shown in Figure 1 and Figure 2. Figure 1A reports the diffraction patterns of LF, LFM05, LFM10, and LFM20; as the Mg-doping level increases, peaks broaden considerably, simultaneously shifting the Bragg angle towards a lower angle. The cell size slightly increases with increasing amounts of Mg^2+^ in agreement with the ionic size of the dopant (Mg^2+^(VI) 0.72 Å vs Fe^3+^(VI) 0.645 Å). The obtained results are in agreement with data from the literature on these system [21].

In Figure 1B, the diffraction patterns of LFC10 and LFG10 compared to the reference LF are reported. In LFC10, the larger ionic radius of Cu^2+^(VI) = 0.73 Å compared to Fe^3+^(VI) counterbalanced the unit cell contraction due to (i) the presence of oxygen vacancies and (ii) redox reactions in the Fe sites (the ionic radius of Fe^4+^(VI) = 0.585 Å) [22]. For LFG10, the shift toward a higher angle indicates a slight decrement of the lattice parameters in agreement with [23].

A shift in the positions of the peaks for LYF10 and LYF20, leading to an overall small reduction in cell volume, can be seen in the enlargement of Figure 2A. The substitution of the La^3+^ ion (1.16 Å) with smaller Y^3+^ (1.019 Å) induced an increase in the octahedral distortion (compression), which led to a reduction in the lattice parameters and unit cell volume, as previously reported [24]. In Figure 2B, LFP03 and LFP05 are reported: the peaks are slightly shifted towards low angles in the diffractograms, corresponding to their cell size increasing with increasing amounts of Pd^2+^. The ionic size of the dopant Pd^2+^ (VI) was 0.86 Å, while that of Fe^3+^ (VI) was 0.645 Å. Palladium oxide (PdO) was detected in the pattern of LFP05, possibly as a result of the small solubility limit of Pd in LaFeO_3_ [25]. However, traces of PdO cannot be excluded in LFP03 if below the detection limit.

### 2.2. Brunauer–Emmett–Teller (BET) Specific Surface Area (SSA)

The specific surface area (SSA) and the crystallite size of the samples are listed in Table 1. With respect to the composition of the reference LF, which shows an SSA of 16 m^2^ g^−1^, the surface areas of LFM05, LFM10, LFM20, and LSF20 were enhanced, reaching a value ~20 m^2^ g^−1^ for LFM20. The crystallite sizes calculated from the XRD analysis ranged from 18 nm (LFM20) to 45 nm (LFG10).

### 2.3. Scanning Electron Microscopy (SEM) and Energy Dispersive X-ray Analysis (EDX)

**Figure 3:**
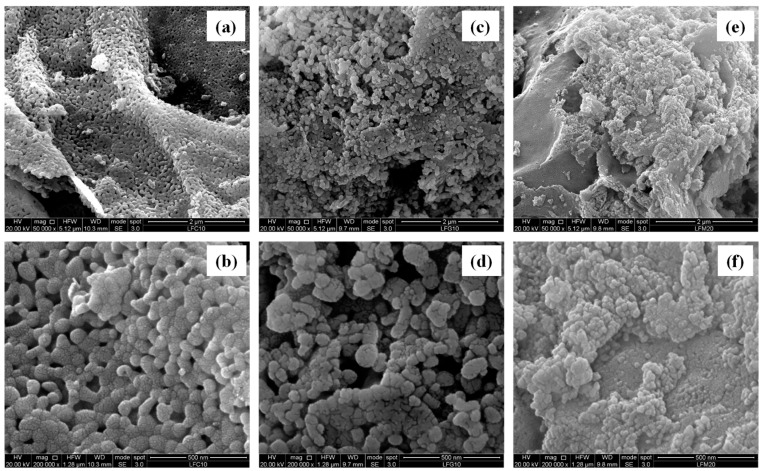
SEM pictures of (**a**,**b**) LFC10; (**c**,**d**) LFG10; and (**e**,**f**) LFM20 at two different magnifications.

**Figure 4:**
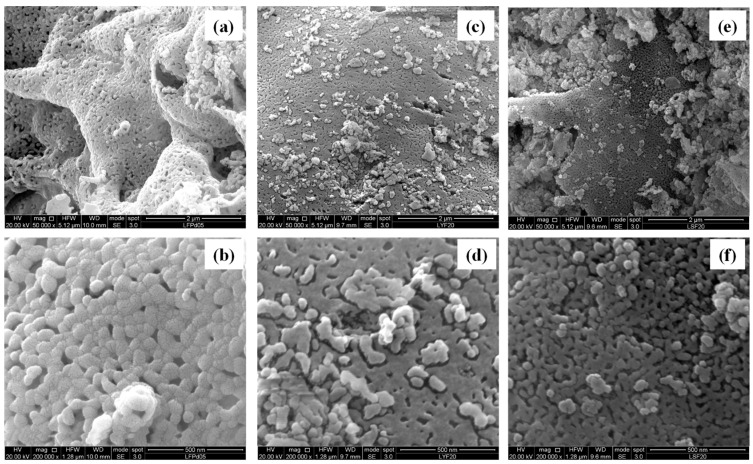
SEM pictures of (**a**,**b**) LFP05; (**c**,**d**) LFY20; and (**e**,**f**) LFS20 at two different magnifications.

The SEM micrographs shown in Figure 3 and Figure 4 are useful for studying the morphology of the investigated samples. In particular, Figure 3 shows images of the LFC10 and LFG10 samples and a micrograph of LFM20, chosen as representative of the three samples containing magnesium. For each sample, two different magnifications (50,000× and 200,000×) are reported. On the other hand, in Figure 4, micrographs of the samples LFP05 and LFY20 (chosen as representative of the samples of their respective series) and those of the LFS20 sample are reported at two different magnifications (50,000× and 200,000×). The morphologies of all the samples studied indicate that, in general, they comprised an agglomerate of particles whose sizes ranged from ca. 20 nm to ca. 100 nm depending on the catalyst. In addition, in all cases, the particles were linked together to form corrugated sheets that were often curved and intertwined, giving rise to the formation of macropores. It is interesting to note that the morphology of the reference LF’s composition, images not reported for the sake of brevity, was similar to that of the substituted LF. By analyzing the various samples in more detail, it can be observed that those that had particles of a smaller size also had a higher specific surface area. In particular, the sample LFM20, which showed the highest SSA (20 m^2^ g^−1^), was the catalyst with the smallest particle size (its average size being in the range of 20–30 nm). On the contrary, the sample LFP05, with a particle size up 100 nm, showed the smallest SSA.

Table 2 reports the percentage of metal content in all of the samples, measured using EDX analysis, compared with that of their nominal compositions. As can be observed in the table, the metal content measured using EDX analysis was generally very close to the nominal one. However, in the case of the LFM20 sample, the amount of magnesium was ca. double with respect to the nominal one, indicating an Mg-rich phase on the surface of the sample, probably due to the high amount of this element in the sample.

### 2.4. Photocatalytic Degradation of CP and OTC

The highly performant photocatalysts on OTC degradation using UV or visible light are listed in Table S1 in Supplementary Materials. Past experimental studies have been generally performed with OTC concentrations in the range 5–50 mg L^−1^, much larger than the one used in this study (0.5 mg L^−1^). Specifically, here, the concentration of the pollutant was chosen taking into account two conditions: (i) the concentration of pollutant measured in surface water is typically <1 μg L^−1^ in different areas [26] and (ii) this concentration must be sufficiently high for spectroscopic detection.

The ability for LF, LFY10, LFC10, and LFM10 to photodegrade CP in aqueous solutions at (i) their natural pH (pH = 6.4) and (ii) in phosphate buffered solutions (pH = 5.0) have been studied. Interestingly, it has been found that the presence of phosphate shifts the maximum absorption peak from λ = 270 nm to λ = 275 nm and decreases the absorption of CP into photocatalysts. Generally, this is ascribed to the chemical characteristics of the antibiotics and their different capabilities for forming chemical complexes. Phosphate absorption can significantly increase the number of negative charges on the photocatalyst surface, causing attraction or repulsion between phosphate and pharmaceuticals, and adsorption-site competition effects can happen due to the smaller molecular size of phosphate ions. The outcomes of our analyses on the degradation of CP are given in Table 3. C_0_ and C_t_ are the concentrations of CP before and after irradiation, respectively. Notably, only 41–22% of CP was found at a natural pH in the presence of the four photocatalysts after 240 min of visible light irradiation, whilst 72–46% of the initial CP was observed after the same irradiation time in the presence of the phosphate buffer. These results suggest that it is important to quantitatively compare the photocatalytic activities of doped-LF using an unbuffered solution for CP.

**Figure 5:**
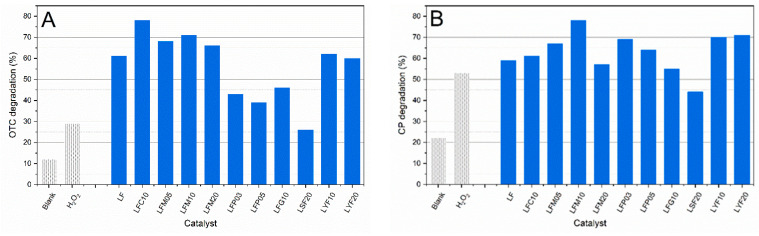
(**A**) Photocatalytic degradation of OTC after 240 min of visible light irradiation. C_0_ = 5 × 10^−6^ M, the OTC solution is phosphate buffered at pH = 5.0, OTC has zwitterionic form. (**B**) Photocatalytic degradation of CP after 240 min of visible light irradiation. C_0_ = 5 × 10^−6^ M, the CP solution is unbuffered (pH = 6.4). Blank: only unbuffered solution of pollutant. H_2_O_2_: solution of pollutant + H_2_O_2_ without photocatalyst.

The photolytic degradation of OTC and CP in neat water are presented in Figure 5A,B (data named “blank”). A number of previous studies have shown that CP is susceptible to direct photochemical transformation from exposure to ultraviolet (UV–A) light [27]. CP photolytically degraded better than OTC, and the same result was found in presence of H_2_O_2_.

Doped-LF-mediated heterogeneous photocatalysis was faster than pure photolysis for both molecules. Previously, it was observed that a small H_2_O_2_ addition is advantageous for the photo-oxidation of CP in the presence of LF [6]. The radical intermediate •OH formed from this oxidant from reactions with the photogenerated electrons can act as an electron scavenger, thus inhibiting the recombination of e^−^/h^+^ pairs at the semiconductor surface [28] according to the following equation:

H_2_O_2_ + e_CB_^−^ → •OH + OH^−^ (1)

where e_CB_^−^ indicates an electron excited to the conduction band.

OTC and CP showed different responses to doped-LF photocatalysts. The most extensive photodegradation of OTC, 78%, was detected with LFC10, and there was also a good response in the presence of the LFM10 photocatalyst. LSF20 showed the lowest degradation value. The most significant outcomes for CP were observed for LFM10, LFY10, and LFY20, with percentages of degradation equal to 78%, 70%, and 71%, respectively. The worst values were found with LSF20, LFG10, and LFM20. Among the doped catalysts, LSF20 showed the worst performance in both CP and OTC degradation. It seems that doping with Sr cancelled the activity of the catalyst; in fact, its activity was similar to that of the test carried out with H_2_O_2_ alone.

To compare the photocatalytic activities of LFM10 and LFC10, the reaction rate constants (k) were calculated using the pseudo first-order model (ln(C_t_/C_0_) versus time) typically used to describe photocatalytic degradation assuming a low initial concentration of the pollutant (Figure S2). As shown in Table 4, the apparent rate constants of OTC were determined to be 0.0051 and 0.0068 min^−1^ for LFM10 and LFC10, respectively, and those of CP were 0.0069 and 0.0042 min^−1^ for LFM10 and LFC10, respectively. Most of the removal percentage of the data in Table S1 are not comparable with ours because UV or solar radiation was used as light source. Few of these studies have been carried out using only radiation in the visible range (entries 3, 7, 8, and 9 in Table S1). Entries 3 and 7 show a very high removal efficiency (and rate constant values greater than those of solar or UV radiation) because they describe studies on composite photocatalysts, in which the enhancement of the photocatalytic performance is related to the presence of the graphene phase that causes a decrement of the recombination of photogenerated electron–hole pairs. Moreover, the unbonded π electrons of graphene provide a large absorption of OTC molecules via *π-π* interactions.

### 2.5. Photocatalytic Degradation of CP and OTC

Generally, the preliminary adsorption of substrate molecules on the surface of a catalyst is necessary for highly efficient photocatalytic degradation [29]. Larger surface areas provide active sites for the adsorption of reagent molecules. The adsorption of molecules on a metal–oxide surface is influenced by the acid-base properties of the surface. Some authors have reported the point of zero charge (PZC) for LF and related oxides. The PVC is a pH of 8.9 for LF [7], around 6.2 for LaFe_0.80_Cu_0.2_O_3_ [30], 7.2 for LaFe_0.9_Co_0.1_O_3_ [31], and 7.2 for La_0.6_Sr_0.4_FeO_3_ [32], suggesting that the surface charge of catalysts is positive when pH < 6.2 as in the case of our experiments. OTC exists predominantly as a cation at pH < 3.6 when the dimethylammonium group is protonated, as a zwitterion between pH 3.6 and 7.5 (resulting from the loss of proton from the phenolic diketone moiety), and as an anion at pH > 7.5 (resulting from the loss of protons from the tricarbonyl system and phenolic diketone moiety) [33]. In this study, the OTC solution was buffered at pH = 5.0, so OTC has zwitterionic form (see Figure S3).

**Figure 6:**
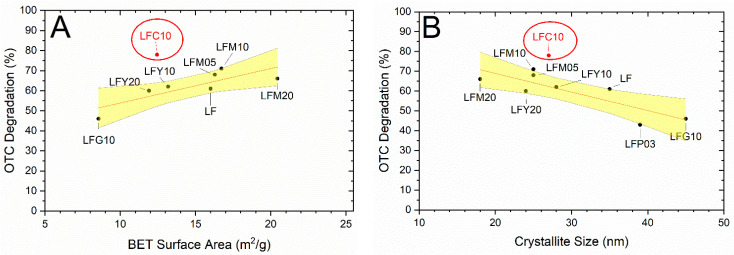
OTC degradation after 240 min as a function of (**A**) BET surface area of the photocatalysts and (**B**) crystallite size calculated via X-ray diffraction measurements. In red is the linear interpolation of the results and in yellow the 95% confidence interval for the interpolation.

Figure 6 shows OTC degradation as a function of the SSA of the photocatalysts (A), and their calculated crystallite size (B). Two catalysts are missing because of the following reasons: (i) LSF20 does not show significant photocatalytic activity (Figure 5A) and (ii) LFP05 is excluded due to the presence of PdO nanoparticles detected from the XRD analysis. In both graphs, the points lay roughly on a straight line, except for the LFC10 photocatalyst. In yellow is the 95% confidence interval for the linear interpolation (in red) of the points. The variation in OTC degradation for doped-LF photocatalysts is most likely the result of an SSA-dependent adsorption on the catalyst surface of the OTC molecules. This relation between the SSA of the photocatalyst and the photocatalytic degradation of OTC indicates that surface reactions are predominant compared to radical reactions in the solution. However, the SSA is not sufficient to explain all results, since the SSA of LFC10 is smaller than, for example, that of LF. Previously, photocatalyst stability was assessed using leaching experiments under solar light in the absence of a pollutant [34]. No significant amounts of Fe, Cu, or La were measured with respect to the lowest limits of their quantification values (Fe = 0.05 mg/L, Cu = 0.03 mg/L and La = 0.1 mg/L). For this reason, a contribution via Cu^2+^ complexation in the solution is excluded. A possible explanation for the better performance of LFC10 could be the presence of oxygen vacancies, as reported in Cu-doped LaAlO_3_, which are favorable for the dissociation of H_2_O_2_ and the generation hydroxyl radicals [35]. As expected, the crystallite size dependence of OTC degradation shows a similar behavior to SSA.

### 2.6. BET Specific Surface Area (SSA) and CP Degradation

**Figure 7:**
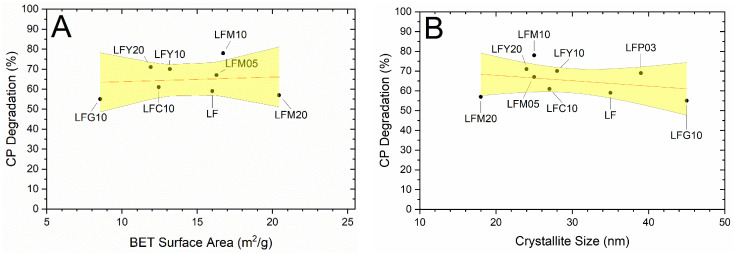
CP degradation after 240 min as a function of (**A**) BET surface area of the photocatalysts and (**B**) crystallite size calculated via X-ray diffraction measurements. The linear interpolation of the results are red lines, and the 95% confidence interval for the interpolation are yellow areas.

CP possesses a carboxylic acid group (pK_a1_ = 6.1) and an amine group in the piperazine moiety (pK_a2_ = 8.7) [36]. It can exist predominantly as a cation at pH < 6.1, as a zwitterion between pH 6.1 and 8.7, and as an anion at pH > 8.7. Surface complexation with positively charged sorbents can occur with deprotonated carboxylate and keto groups. Figure 7 shows CP degradation as a function of the SSA of the photocatalysts and their calculated crystallite size. Unlike what is observed with OTC, the results suggest that microstructural features have poor influence on the extent of the degradation. In this study, the initial pH of the solution was about 6.4, so CP zwitterionic and cationic forms were predominant. During the degradation reactions, the pH dropped just below 6.1 due to the formation of inorganic acids, such as HF and HNO_3_, and low molecular weight organic species, such as carboxylic acids [37]. In general, pH affected the adsorption of the pollutant on the catalyst. When the solution pH was lower than 6.1, the surface of the doped-LF oxides was positively charged, and the catalyst exhibited electrical repulsion that hindered the adsorption of CP cations; thus, the reaction efficiency dropped. This fact is evident from an analysis of the results obtained from the photocatalytic tests performed with CP at a pH of 5 (see Table 3). The degradation of CP observed at a pH of 5 indicates that the reaction occurred only in the homogeneous phase and, furthermore, that the presence of the catalyst negatively affected the degradation of CP as a result of a light shielding effect. In fact, the degradation rate of CP in the presence of a photocatalyst was always lower than that of CP in the presence of H_2_O_2_ alone. On the contrary, the tests carried out at an initial pH of 6.4 indicate that the degradation of CP in the presence of a photocatalyst was generally slightly higher than that of CP in the presence of H_2_O_2_ alone. This indicates that the homogeneous reaction is the prevailing one and, therefore, at this pH, a leveling in the amount of CP degraded is observed.

## 3. Materials and Methods

### 3.1. Chemicals

All the chemicals, solvents, and reagents used in this study were purchased from Sigma–Aldrich Europe (Milano, Italy) and used without purification. Ciprofloxacin (≥98%) and Oxytetracycline dyhydrate (≥97%) were used as model pollutants. Lanthanum ferrite LaFeO_3_ (LF), 10 mol% Cu-doped LF (LFC10), 5 mol% Mg-doped LF (LFM05) 5), 10 mol% Mg-doped LF (LFM10), 20 mol% Mg-doped LF (LFM20), 10 mol% Ga-doped LF (LF G10), 3 mol% Pd-doped LF (LFP03), 5 mol% Pd-doped LF (LFP05), 10% mol Y-doped LF (LFY10), 20 mol% Y-doped LF (LFY20), and 20 mol% Sr-doped LF (LFS20) nanopowders were prepared using the citrate auto-combustion method, as described in our previous work [22].

### 3.2. Microstructural Characterization

The powder X-ray diffraction patterns of the catalysts were recorded using a Bruker D8-Advance powder diffractometer equipped with a Cu kα X-ray source and a Lynxeye XE-T^®^ solid-state detector. The patterns were recorded in an interval of 10–90° 2θ with a step of 0.01° and a counting time of 1 s per step. X-ray data were used to calculate domain size using the Scherrer equation. Brunauer–Emmett–Teller (BET) specific surface area (SSA) determination was performed with nitrogen absorption on about 500 mg of the samples using a Micrometric Tristar 3000 automated gas-adsorption analyzer.

### 3.3. Scanning Electron Microscopy (SEM) and Energy Dispersive X-ray Analysis (EDX)

Scanning electron microscopy (SEM) was performed using an FEI Quanta 200 ESEM microscope, operating at 20 kV on specimens from which a thin layer of gold had evaporated. On the other hand, an electron microprobe used in an energy dispersive mode (EDX) was employed to obtain information on the actual metal-content ratio present in the samples.

### 3.4. Photocatalytic Studies

Photocatalytic degradation reactions were carried out using a Ryonet reactor equipped with 6 fluorescent lamps (daylight, 8W, GE lighting 10055-F8T5/D) emitting in the 380–780 nm region. The temperature was kept constant at 28 ± 1 °C through a liquid cooling system. In the typical process, 2.6 mg of photocatalyst powder was added to 20 mL of a 5.0 × 10^−6^ M aqueous solution of CP or OTC with the presence of 10^−2^ M H_2_O_2_. The mixture was stirred in the dark for 20 min in a Pyrex glass tube in order to allow for an adsorption/desorption equilibrium on the catalyst surface. Then, the 6 lamps were turned on for 4 h at different time intervals (t = 0, 30, 60, 120, and 240 min), and aliquots of the reacting suspension (2.5 mL) were taken. The samples were centrifugated for 5 min at 350 rpm (Scharlab BL-8), and their supernatants were analyzed on the spectrophotometer (see Section 2.5). The photocatalytic activities of the samples were evaluated by monitoring the degradation of CP in a buffered solution (buffer phosphate, 100 mM pH = 5.0) and in an unbuffered CP solution (initial solution pH = 6.0, and about 6.4 when the photocatalysts were also present). The degradation of OTC was evaluated only in the buffered solution (buffer phosphate, 100 mM pH = 5.0) since, at pH 5.0, OTC cannot be photolyzed under visible light because of its poor visible light absorption [38]. All degradation tests were repeated twice.

### 3.5. UV–Vis Spectroscopy

**Figure 8:**
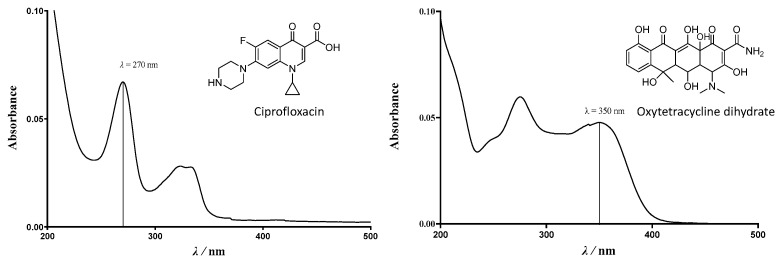
UV–Vis absorption spectra of ciprofloxacin and oxytetracycline.

The absorption spectra were measured using an ultraviolet–visible (UV–Vis) double-beam spectrophotometer (Jasco V-650) with a 10-mm light path and quartz cuvettes. Full spectra were taken in order to monitor any spectral change that may have occurred. The degradation of CP and OTC were calculated using the formula C_t_/C_0_, where C_0_ and C_t_ are the concentrations of the pollutant in the solution before and after irradiation, respectively, at a set time (t). Degradation values are the average of two independent assays. The main absorption peaks were detected at 270 nm for CP and 350 nm for OTC. Figure 8 shows the absorption spectra of the investigated molecules.

## 4. Conclusions

Many studies focusing on heterogeneous photocatalysis for water treatment have reported on the synthesis of novel semiconductor oxides, their physico-chemical characterizations, and the degradation of model compounds. We have shown herein that the selection of the substrate (model compound) is critical. To obtain reliable conclusions, it is good to use one (or more) model molecules exhibiting electrostatic attraction with the surface of the photocatalyst and also one exhibiting electrostatic repulsion.

In this study, pure and doped semiconductor oxides (La_1-x_A_x_FeO_3_ A = Y; Sr and LaFe_1-x_B_x_O_3_ B = Cu, Ga, Mg, Pd) were prepared using the sol-gel method and used for the photocatalytic degradation of model pollutants, the OTC and CP antibiotics in water. LFM10 and LFC10 are able to remove 71% and 78% of OTC (C_0_ = 5 × 10^−6^ M, buffer pH = 5.0) in 240 min in the presence of H_2_O_2_ and under visible light irradiation, respectively. The specific surface area (SSA) and doping type are the main factors affecting degradation capacity.

LFM10, LFY10, and LFY20 photocatalysts showed a remarkable photocatalytic activity degrading CP (78%, 71%, and 70% of CP removal in 240 min, respectively) using starting conditions C_0_ = 5 × 10^−6^ M and a natural pH of 6.4. OTC and CP exist predominantly as a cation at pH < 3.6 and pH < 6.1, respectively. As a consequence, unlike what is observed with OTC, when the electrostatic repulsion between the pollutant (CP) and the oxide surface is predominant (pH below 6.1), the microstructural features have poor influence on the extent of degradation.

## Figures and Tables

**Table 1:** BET specific surface area (SSA) and crystallite size (calculated using Scherrer equation) of substituted LaFeO_3_.

Sample	Nominal Composition	BET (m^2^ g^−1^)	Crystallite Size (nm)
LFC10	LaFe_0.90_Cu_0.10_O_3_	12	27
LFG10	LaFe_0.90_Ga_0.10_O_3_	9	45
LFM05	LaFe_0.95_Mg_0.05_O_3_	16	25
LFM10	LaFe_0.90_Mg_0.10_O_3_	17	25
LFM20	LaFe_0.80_Mg_0.20_O_3_	20	18
LFP03	LaFe_0.97_Pd_0.03_O_3_	n.a.	39
LFP05	LaFe_0.95_Pd_0.05_O_3_	n.a.	31
LFS20	La_0.80_Sr_0.20_FeO_3_	17	26
LFY10	La_0.90_Y_0.10_FeO_3_	13	28
LFY20	La_0.80_Y_0.20_FeO_3_	12	24

**Table 2:** EDX and nominal (in parenthesis) atomic metal percentages.

Sample	La	Fe	Cu	Mg	Ga	Pd	Y	Sr
LFC10	49 (50)	46 (45)	5.0 (5.0)	-	-	-	-	-
LFM05	50 (50)	47 (47.5)	-	3.0 (2.5)	-	-	-	-
LFM10	46 (50)	49 (45)	-	5.0 (5.0)	-	-	-	-
LFM20	45 (50)	38 (40)	-	17 (10)	-	-	-	-
LFG10	50 (50)	44 (45)	-	-	6.0 (5.0)	-	-	-
LFP03	46 (50)	52 (48.5)	-	-	-	2.0 (1.5)	-	-
LFP05	49 (50)	48 (47.5)	-	-	-	3.0 (2.5)	-	-
LFY10	45 (45)	50 (50)	-	-	-	-	5.0 (5.0)	-
LFY20	39 (40)	49 (50)	-	-	-	-	12 (10)	-
LFS20	40 (40)	50 (50)	-	-	-	-	-	10 (10)

**Table 3:** Photocatalytic degradation results reported as residual percentage of CP (calculated as C_t_/C_0_∙100) after 240 min of visible light irradiation in unbuffered solution of pollutant (C_0_ = 5 × 10^−6^ M, pH = 6.4) and in phosphate buffered solution (pH = 5.0).

Solution	LF	LFY10	LFC10	LFM10
CP unbuffered	41	30	39	22
CP buffered	62	55	72	46

**Table 4:** The pseudo first order rate constants of OTC and CP degradation from LFM10 and LFC10 at room temperature under various conditions.

Catalyst	Catalyst Loading (mg L^−1^)	Pollutant	Pollutant (mol L^−1^)	Additive	*k*_1_ (min^−1^)	*R* ^2^
LFM10	130	OTC	5 × 10^−6^ M	H_2_O_2_	0.0051	0.999
LFC10	130	OTC	5 × 10^−6^ M	H_2_O_2_	0.0068	0.980
LFM10	130	CP	5 × 10^−6^ M	H_2_O_2_	0.0069	0.988
LFC10	130	CP	5 × 10^−6^ M	H_2_O_2_	0.0042	0.951

## Data Availability

Data is contained within the article or supplementary material.

## Supplementary Materials

The following supporting information can be downloaded at: https://www.mdpi.com/article/10.3390/molecules28093807/s1. Figure S1: SEM picture of LF; Figure S2: Kinetics for the photocatalytic degradation of OTC (5 × 10^−6^ M) in the presence of LFC10 and LFM10 photocatalysts with the presence of 10^−2^ M H_2_O_2_; Figure S3: Ionization of OTC at various pH; Table S1: Comparison of the experimental conditions for degradation of OTC. For comparison, OTC photolysis results are also shown. References [38,39,40,41,42,43,44,45,46,47] are cited in Supplementary Materials.
