# Supplementary material for: Comparison of Ten Metal-Doped LaFeO3 Samples on Photocatalytic Degradation of Antibiotics in Water under Visible Light: Role of Surface Area and Aqueous Phosphate Ions
Source: Molecules. 2023 Apr 29;28(9):3807. doi: 10.3390/molecules28093807 (PMC10179954; doi:10.3390/molecules28093807)
Supplement: Supplementary file 1 [file molecules-28-03807-s001.zip › molecules-2354246-supplementary.pdf]

molecules

MDPI

Comparison of Ten Metal-Doped $LaFeO_3$ Samples on Photocatalytic Degradation of Antibiotics in Water under Visible Light: Role of Surface Area and Aqueous Phosphate Ions

Isabella Bolognino [1], Renato Pelosato [1], Giuseppe Marcì [2] and Isabella Natali Sora [1,*]

[1] Department of Engineering and Applied Sciences and INSTM, University of Bergamo, Viale Marconi 5, 24044 Dalmine, Italy; isabella.bolognino@unibg.it (I.B.); renato.pelosato@unibg.it (R.P.)
[2] "Schiavello Grillone" Photocatalysis Group, Department of Engineering, University of Palermo, Viale delle Scienze, 90128 Palermo, Italy; giuseppe.marci@unipa.it
* Correspondence: isabella.natali-sora@unibg.it

Supporting Information

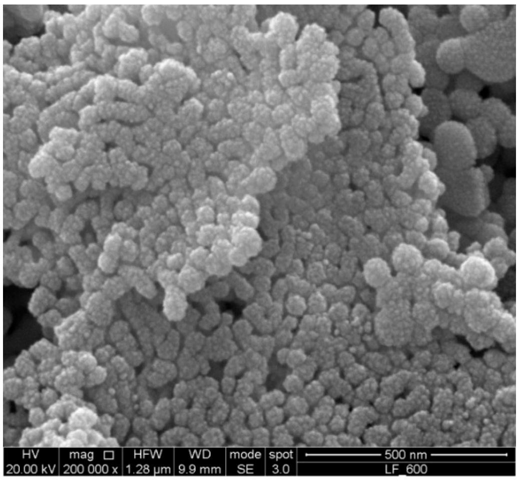

Figure S1. SEM picture of LF.

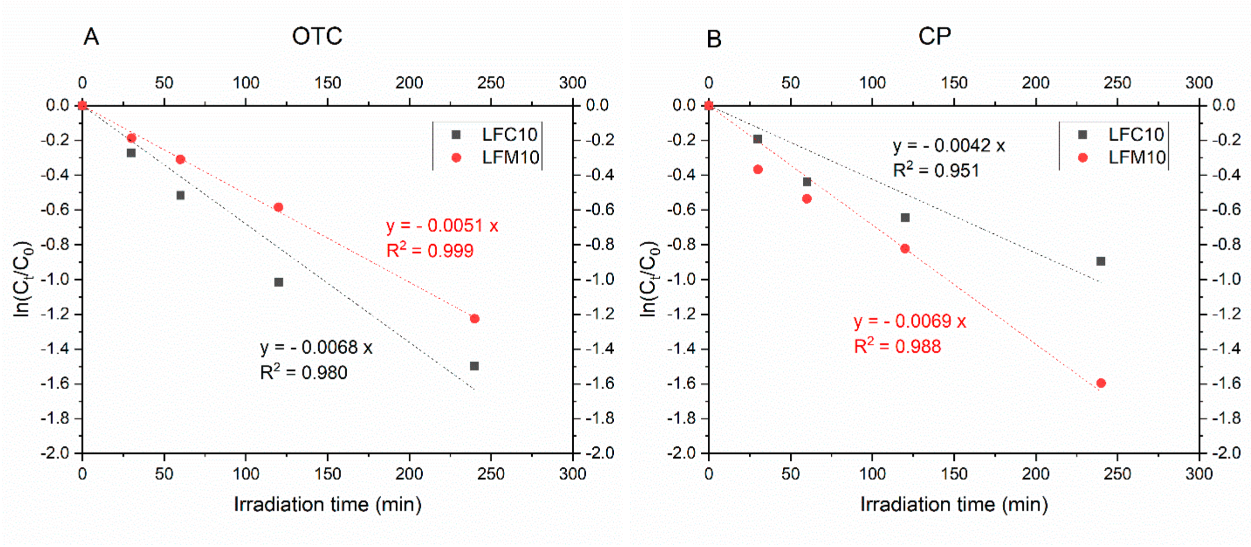

Figure S2. Kinetics for the photocatalytic degradation of OTC ($5{\cdot}10^{-6}$ M) in the presence of LFC10 and LFM10 photocatalysts with the presence of 10^{-2} M H_2O_2.

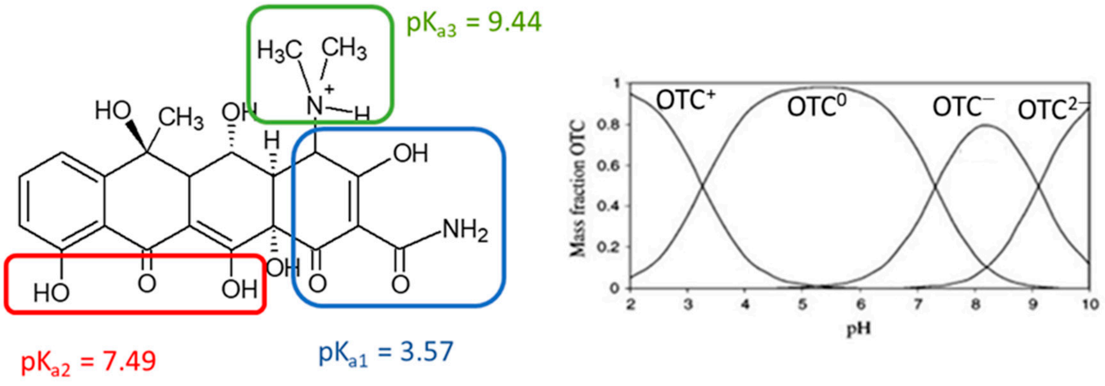

Figure S3. Ionization of OTC at various pH [1].

Table S1: Comparison of the experimental conditions for degradation of OTC. For comparison also OTC photolysis results are shown.

Entry	Photocatalyst	Light source	OTC concentration	Catalyst concentration	Removal %	Time (min)	*k* (min^{-1})	Ref
**1**	(Photolysis)	UV Hg 500 W	10 mg/L	–	95	240	0.0141	[2]
**2**	Ti-MCM-41	UV Hg 100 W	50 mg/L	1 g/L	87	180	0.012	[3]
**3**	Graphene/TiO2/ZSM5	Visible 300 W	10 mg/L	0.2 g/L	100	180	0.04	[4]
**4**	Co_3O_4/TiO_2/GO	(311-600 nm) Xe 300 W	10 mg/L	0.25 g/L	91	90	0.0272	[5]
**5**	TiO_2	Simulated solar Xe 1000 W	20 mg/L	0.5g/L	100	40	?	[6]
**6**	graphitic C_3N_4/ $NiFe_2O_4$	Solar radiation	50 mg/L	0.5 g/L	97	60	0.036	[7]
**7**	$Fe_{2.8}Ce_{0.2}O_4$/GO	Visible 220 W	30 mg/L	0.8 g/L	78	120	0.0119	[8]
**8**	2wt% $LaFeO_3$/g-C_3N_4 nanosheets	Visible light 40W white LED	40 mg/L	-	90	120	Second order	[9]
**9**	N and F doped TiO_2 film	Visible light	5 mg/L	-	70	420	-	[10]

References

1. Kulshrestha, P.; Giese Jr., R.F.; Aga, D.S. Investigating the Molecular Interactions of Oxytetracycline in Clay and Organic Matter: Insights on Factors Affecting Its Mobility in Soil. *Environmental Science and Technology* **2004**, *38*, 4097–4105, doi:10.1021/es034856q.
2. Jiao, S.; Zheng, S.; Yin, D.; Wang, L.; Chen, L. Aqueous Oxytetracycline Degradation and the Toxicity Change of Degradation Compounds in Photoirradiation Process. *Journal of Environmental Sciences* **2008**, *20*, 806–813, doi:10.1016/S1001-0742(08)62130-0.
3. Chen, H.; Peng, Y.-P.; Chen, K.-F.; Lai, C.-H.; Lin, Y.-C. Rapid Synthesis of Ti-MCM-41 by Microwave-Assisted Hydrothermal Method towards Photocatalytic Degradation of Oxytetracycline. *Journal of Environmental Sciences (China)* **2016**, *44*, 76–87, doi:10.1016/j.jes.2015.08.027.
4. Hu, X.-Y.; Zhou, K.; Chen, B.-Y.; Chang, C.-T. Graphene/TiO 2 /ZSM-5 Composites Synthesized by Mixture Design Were Used for Photocatalytic Degradation of Oxytetracycline under Visible Light: Mechanism and Biotoxicity. *Applied Surface Science* **2016**, *362*, 329–334, doi:10.1016/j.apsusc.2015.10.192.
5. Jo, W.-K.; Kumar, S.; Isaacs, M.A.; Lee, A.F.; Karthikeyan, S. Cobalt Promoted TiO2/GO for the Photocatalytic Degradation of Oxytetracycline and Congo Red. *Applied Catalysis B: Environmental* **2017**, *201*, 159–168, doi:10.1016/j.apcatb.2016.08.022.
6. Pereira, J.H.O.S.; Vilar, V.J.P.; Borges, M.T.; González, O.; Esplugas, S.; Boaventura, R.A.R. Photocatalytic Degradation of Oxytetracycline Using TiO2 under Natural and Simulated Solar Radiation. *Solar Energy* **2011**, *85*, 2732–2740, doi:10.1016/j.solener.2011.08.012.
7. Sudhaik, A.; Raizada, P.; Shandilya, P.; Singh, P. Magnetically Recoverable Graphitic Carbon Nitride and NiFe2O4 Based Magnetic Photocatalyst for Degradation of Oxytetracycline Antibiotic in Simulated Wastewater under Solar Light. *Journal of Environmental Chemical Engineering* **2018**, *6*, 3874–3883.
8. Hassandoost, R.; Pouran, S.R.; Khataee, A.; Orooji, Y.; Joo, S.W. Hierarchically Structured Ternary Heterojunctions Based on Ce3+/ Ce4+ Modified Fe3O4 Nanoparticles Anchored onto Graphene Oxide Sheets as Magnetic Visible-Light-Active Photocatalysts for Decontamination of Oxytetracycline. *Journal of Hazardous Materials* **2019**, *376*, 200–211.
9. Xu, K.; Yang, X.; Ruan, L.; Qi, S.; Liu, J.; Liu, K.; Pan, S.; Feng, G.; Dai, Z.; Yang, X.; et al. Superior Adsorption and Photocatalytic Degradation Capability of Mesoporous LaFeO3 /g-C3 N4 for Removal of Oxytetracycline. *Catalysts* **2020**, *10*.
10. Zhao, C.; Pelaez, M.; Duan, X.; Deng, H.; O'Shea, K.; Fatta-Kassinos, D.; Dionysiou, D.D. Role of PH on Photolytic and Photocatalytic Degradation of Antibiotic Oxytetracycline in Aqueous Solution under Visible/Solar Light: Kinetics and Mechanism Studies. *Applied Catalysis B: Environmental* **2013**, *134–135*, 83–92.
